# Focus on the Role of the NLRP3 Inflammasome in Multiple Sclerosis: Pathogenesis, Diagnosis, and Therapeutics

**DOI:** 10.3389/fnmol.2022.894298

**Published:** 2022-05-25

**Authors:** Yueran Cui, Haiyang Yu, Zhongqi Bu, Lulu Wen, Lili Yan, Juan Feng

**Affiliations:** Department of Neurology, Shengjing Hospital of China Medical University, Shenyang, China

**Keywords:** inflammation, NLRP3 inflammasome, multiple sclerosis, biomarker, treatment

## Abstract

Neuroinflammation is initiated with an aberrant innate immune response in the central nervous system (CNS) and is involved in many neurological diseases. Inflammasomes are intracellular multiprotein complexes that can be used as platforms to induce the maturation and secretion of proinflammatory cytokines and pyroptosis, thus playing a pivotal role in neuroinflammation. Among the inflammasomes, the nucleotide-binding oligomerization domain-, leucine-rich repeat- and pyrin domain-containing 3 (NLRP3) inflammasome is well-characterized and contributes to many neurological diseases, such as multiple sclerosis (MS), Alzheimer's disease (AD), and ischemic stroke. MS is a chronic autoimmune disease of the CNS, and its hallmarks include chronic inflammation, demyelination, and neurodegeneration. Studies have demonstrated a relationship between MS and the NLRP3 inflammasome. To date, the pathogenesis of MS is not fully understood, and clinical studies on novel therapies are still underway. Here, we review the activation mechanism of the NLRP3 inflammasome, its role in MS, and therapies targeting related molecules, which may be beneficial in MS.

## Introduction

Neuroinflammation is caused by innate immune response; however, the dysregulation of innate immunity can be deleterious to the nervous system (Leite et al., 2022). It has been found that inflammasomes participate in innate immunity and link innate immunity with inflammation (Awad et al., 2018). Inflammasomes are cytosolic multiprotein platforms that exert their effects through pattern-recognition receptors (PRRs), thus inducing the maturation and secretion of proinflammatory cytokines interleukin-1β (IL-1β) and interleukin-18 (IL-18) (Kayagaki et al., 2015). PRRs form inflammasomes and act as sensors. To date, there are a few known PRRs, such as nucleotide-binding oligomerization domain-like receptor family members NLRP1, NLRP3, NLRC4, absent-in-melanoma 2 (AIM2), and pyrin, and they sense different kinds of stimuli (Broz and Dixit, 2016). Among inflammasomes, the nucleotide-binding oligomerization domain-, leucine-rich repeat-, and pyrin domain-containing 3 (NLRP3) inflammasome is reported to sense a wide range of stimuli and involve in a variety of diseases.

**Figure 1:**
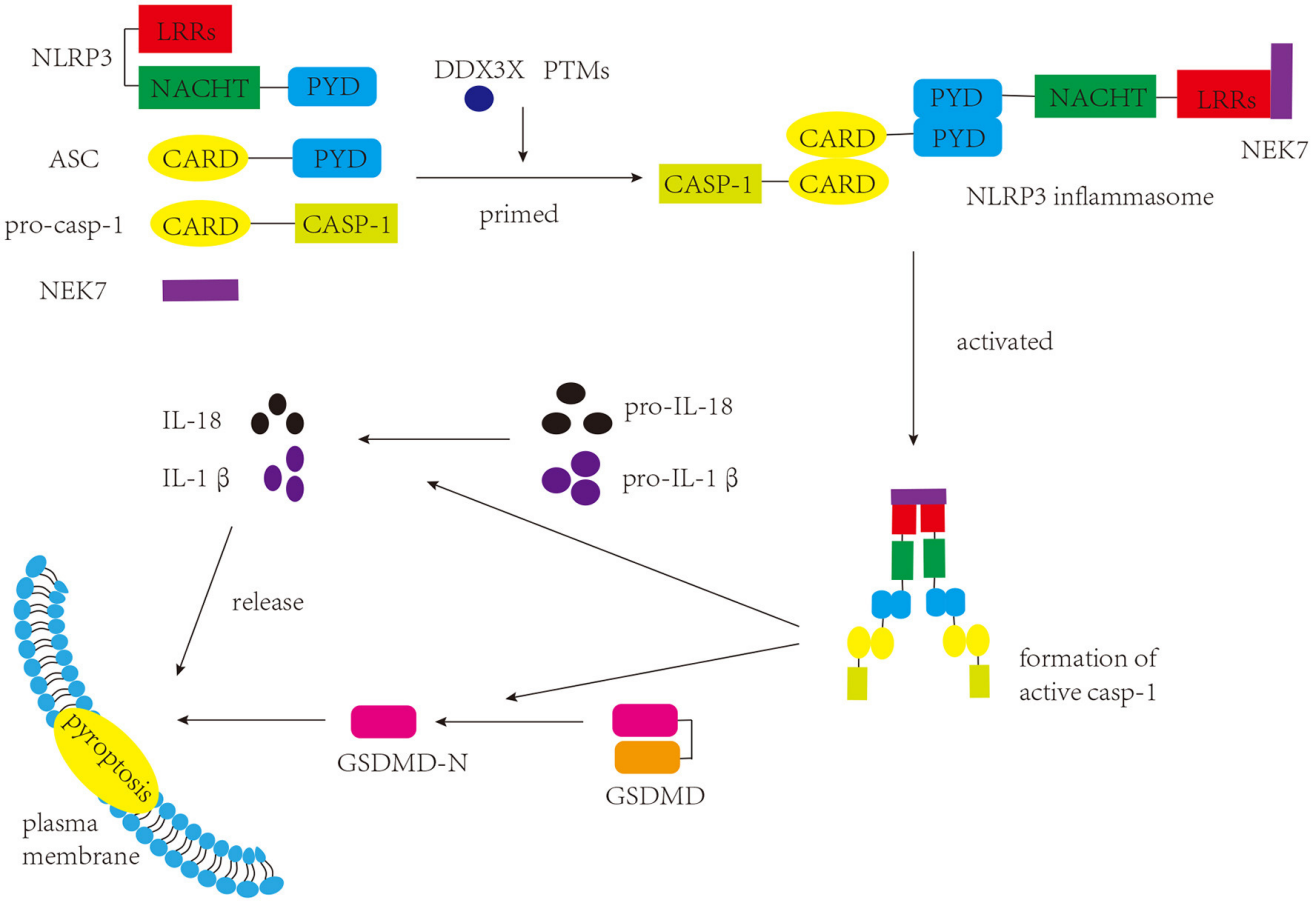
Components and function of NLRP3 inflammasome. The NLRP3 inflammasome consists of three components: NLRP3, ASC, and pro-caspase-1. Once stimulated, the assembly and activation of the NLRP3 inflammasome lead to the production of active caspase-1. Caspase-1 cleaves GSDMD to produce GSDMD-N, which interacts with the plasma membrane and induces pyroptosis. Caspase-1 can also promote the maturation of pro-IL-1β and pro-IL-18 into IL-1β and IL-18, respectively, which are either secreted or released during pyroptosis.

NLRP3 inflammasome was initially characterized in Muckle-Wells syndrome, which is an autoinflammatory disease (Martinon et al., 2002). As a common inflammasome, the NLRP3 inflammasome senses a wide range of danger- and pathogen-associated molecular patterns (DAMPs and PAMPs, respectively), and participates in a myriad of immune and inflammatory diseases (Peñin-Franch et al., 2022). There are three components of the NLRP3 inflammasome: NLRP3 protein (sensor), apoptosis-associated specklike protein containing a caspase-activation and recruitment domain (ASC) (adaptor), and pro-caspase-1 (effector) (Broz and Dixit, 2016) (Figure 1). NLRP3 protein itself comprises three domains: a C-terminal leucine-rich repeats (LRRs) domain, a central nucleotide binding and oligomerization domain (NACHT), and an N-terminal pyrin domain (PYD) (Kumar et al., 2011). Under normal conditions, LRRs and NACHT domains bind to each other, and NLRP3 protein is unable to bind ASC (Shao et al., 2015). Once stimulated, the two domains separate, and the NLRP3 protein binds to ASC *via* the PYD. ASC has two domains: a C-terminal caspase activation and recruitment domain (CARD) and an N-terminal PYD (Dick et al., 2016). When interacting with the NLRP3 protein, ASC can bind to pro-caspase-1 through the CARD, promoting the production of cleaved caspase-1, which in turn induces the maturation and release of IL-1β and IL-18 and the occurrence of pyroptosis (Shao et al., 2015; Liu et al., 2016). Pyroptosis, a form of programmed cell death that differs from apoptosis and necrosis, induces membrane rupture, leading to the release of inflammatory factors (Shi et al., 2017).

Multiple sclerosis (MS) is a common autoimmune, neurodegenerative disease of the central nervous system (CNS) (Calahorra et al., 2022). More than two million people around the world suffer from it, which brings a huge social and economic burden to patients (Browne et al., 2014; Kobelt et al., 2017). Clinically, MS is classified into four phenotypes: clinically isolated syndrome, primary progressive MS, secondary progressive MS, and progressive relapsing MS. There is no clear distinction among the different phenotypes. MS diagnosis requires a combination of clinical manifestations, imaging studies, and laboratory tests. Among the diagnostic techniques, MRI is very important (Brownlee et al., 2015). The clinical manifestations of MS patients are diverse and mainly include ataxia, cognitive dysfunction, and visual impairment (Pinke et al., 2020), which may be related to the complexity of its etiology. Currently, the exact etiology of MS is unclear and research has proposed several possibilities, which include genetic susceptibility and single nucleotide polymorphisms (SNP) of immune system-related genes, Epstein-Barr virus (EBV) infection, smoking, and reduced levels of Vitamin D (Hafler et al., 2007; Healy et al., 2009; Endriz et al., 2017; de Oliveira et al., 2020). In addition, dysfunction of the intestinal flora, a research hotspot in recent years, has also been reported to be related to MS (Pröbstel and Baranzini, 2018). According to studies, myelin-specific autoreactive CD4+ T cells may be activated in the periphery during early stages of MS. CD4+ T cells, B cells, and macrophages cross the blood-brain barrier and migrate into the CNS after destruction of the blood-brain barrier. Furthermore, antigen-presenting cells (APCs) induce differentiation of CD4+ T cells into the Th1 and Th17 cells, which exacerbate inflammatory response and contribute to the progression of MS (Shao et al., 2021). As the disease progresses, the infiltration of peripheral immune cells decreases, whereas neurodegenerative changes increase in the brain (Serafini et al., 2004; Rottlaender and Kuerten, 2015). Currently, clinical treatment for MS is not ideal. Disease-modifying therapies have been shown to be beneficial to MS patients and usually focus on recovery from relapses and slowing the progression. However, these therapies have serious side effects and do not inhibit the progressive increase in neurological disability. Therefore, more research is needed to develop effective treatment for MS patients.

## Mechanisms of NLRP3 Inflammasome Activation

**Figure 2:**
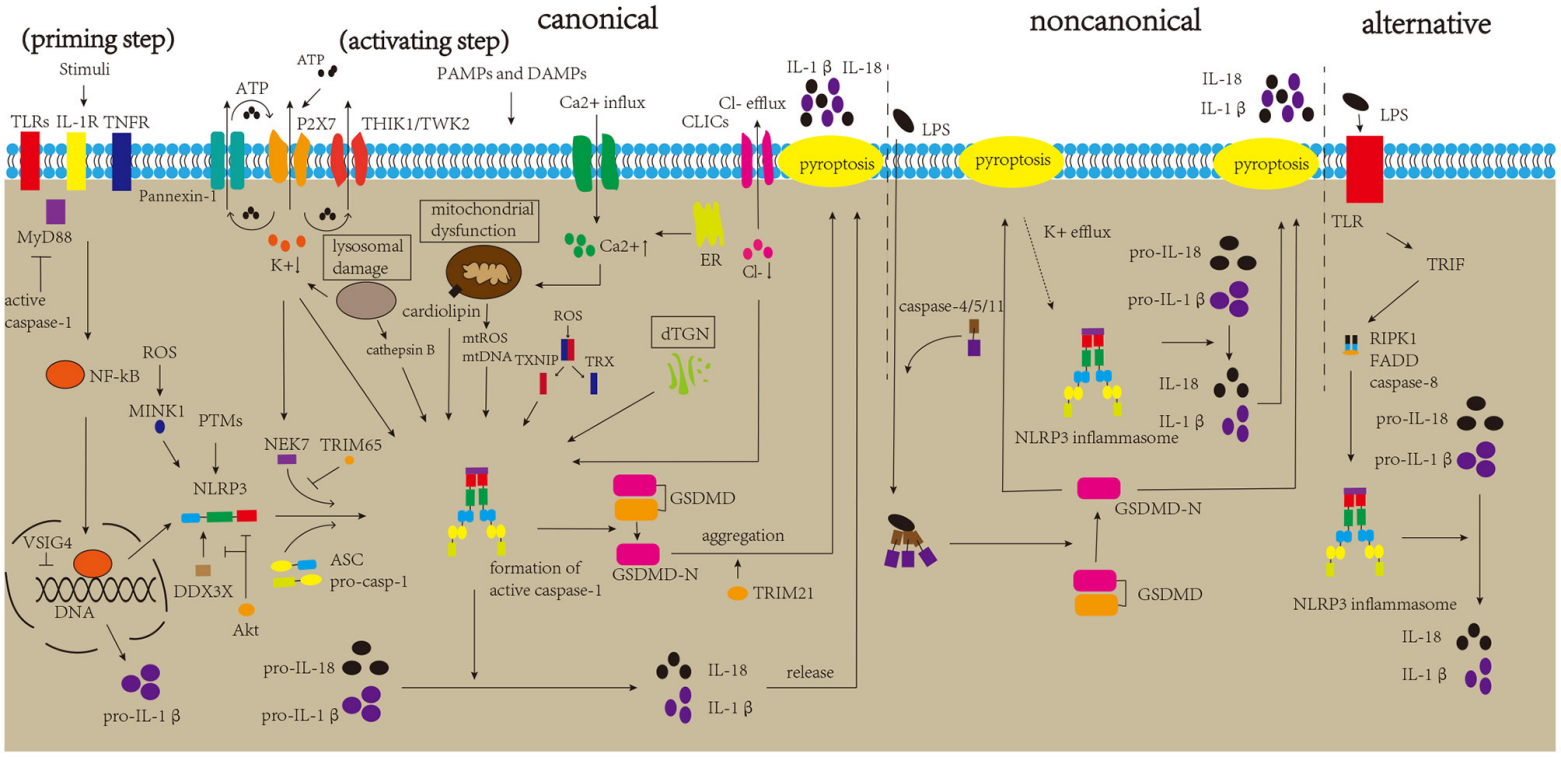
Mechanisms of NLRP3 inflammasome activation. The canonical activation pathway of the NLRP3 inflammasome includes priming and activating step. In the priming step, stimulatory factors can bind to TLRs and other molecules, promoting the production of NLRP3 and pro-IL-1β. After priming, DAMPs and PAMPs can generate major activation signals, such as ion fluctuations, mitochondrial dysfunction, lysosomal damage, and dTGN, which affect the assembly and activation of the NLRP3 inflammasome. The activated NLRP3 inflammasome triggers the maturation of pro-caspase-1 into active caspase-1. Caspase-1 can induce the maturation and release of IL-1β and IL-18 and pyroptosis, thereby aggravating neuroinflammation. The non-canonical pathway is mediated by caspase-4/5/11, which directly sense cytosolic LPS, and K+ efflux due to pyroptosis can trigger NLRP3 inflammasome activation. The alternative pathway is mediated by the TLR4-TRIF-RIPK1-FADD-caspase-8 signaling pathway, triggering NLRP3 inflammasome activation.

NLRP3 inflammasome is the most widely studied inflammasome. However, the mechanism underlying its activation remains to be elucidated. The canonical activation pathway includes a priming step (signal 1) and an activating step (signal 2) (Figure 2) (Bauernfeind et al., 2009; Kelley et al., 2019). In the priming step, stimuli interact with the Toll-like receptor (TLR)-adaptor molecule myeloid differentiation primary response 88 (MyD88), interleukin-1 receptor (IL-1R) and/or cytokine receptors, such as the tumor necrosis factor receptor (TNFR), thus activating the nuclear factor kappa B (NF-kB) pathway, which increases the expression levels of NLRP3 protein and pro-IL-1β (Latz et al., 2013). Post-translational modifications (PTMs) of NLRP3 protein are crucial to NLRP3 inflammasome activation. Once the NLRP3 protein is stimulated by signal 2, it begins to undergo deubiquitination and phosphorylation, which allows it to oligomerize and form the NLRP3 inflammasome (He et al., 2016a; Jo et al., 2016). In the activating step, many DAMPs and PAMPs can promote the assembly and activation of the NLRP3 inflammasome, which causes the formation of cleaved caspase-1, inducing the release of IL-1β and IL-18 and pyroptosis (Kayagaki et al., 2015; Shi et al., 2015; Liu et al., 2016). Several regulatory mechanisms are involved in the canonical activation pathway. For example, VSIG4 is reported to mediate the inhibition of *Nlrp3* and *Il-1*β at the transcriptional level (Huang et al., 2019). In addition, NEK7 and DDX3X are thought to bind to the NLRP3 protein, which is crucial for NLRP3 inflammasome formation. DDX3X is an ATPase/RNA helicase of the DEAD-box family that directly binds to the NLRP3 protein. Research has reported that the knockdown of *Ddx3x* in peritoneal macrophages inhibits NLRP3 inflammasome activation and pyroptosis (Samir et al., 2019). Studies found that AKT could block the interaction between DDX3X and NLRP3 by phosphorylating DDX3X. Alternatively, AKT could also directly interact with NLRP3, thus inhibiting NLRP3 oligomerization (Zhao et al., 2020; Guo et al., 2021). NEK7 binds and bridges the NLRP3 protein, triggering NLRP3 inflammasome activation, which is affected by the status of NLRP3 S803, a site that is phosphorylated after priming and later dephosphorylated after activation (Sharif et al., 2019; Niu et al., 2021). A recent study showed that TRIM65, an E3 Ubiquitin Ligase, can interact with the NACHT domain of NLRP3 and promote ubiquitination of lys48 and lys63 of NLRP3, thus inhibiting the NEK7-NLRP3 interaction and suppressing the activation of NLRP3 inflammasome (Tang et al., 2021). In addition, misshapen (Msn)/NIK-related kinase 1 (MINK1) interacts with the NLRP3 LRR domain, inducing the phosphorylation of Ser725 and priming the activation of the NLRP3 inflammasome (Zhu et al., 2021). Moreover, research has demonstrated a negative feedback regulation between caspase-1 and MyD88, which may suggest self-regulation of the body (Avbelj et al., 2021). NLRP3 inflammasome activation can promote the formation of GSDMD-N, inducing pyroptosis. Gao et al. reported that TRIM21, a tripartite motif protein, binds to GSDMD *via* its PRY-SPRY domain, and positively regulates GSDMD-dependent pyroptosis. It maintains stable expression of GSDMD in resting cells, while inducing GSDMD-N aggregation during pyroptosis (Gao et al., 2022).

Several stimuli can activate the NLRP3 inflammasome, and it is impossible for NLRP3 inflammasome to directly bind to the stimulatory factors. These stimuli may generate major activation signals, triggering activation of the NLRP3 inflammasome. Many related studies have proposed some major activation signals, such as ion fluctuations, dysfunction of mitochondria, lysosomal disruption, and Golgi dispersal (Song et al., 2017; Shao et al., 2021). This is discussed in detail in the following sections.

The ion fluctuations involve K+, Ca2+, and Cl–. There is copious extracellular ATP due to tissue damage and immune cells activation during inflammation, and high concentrations of extracellular ATP activate P2X7R, which induces K+ efflux and then recruits Pannexin-1 to release intracellular ATP (Gombault et al., 2012; Bartlett et al., 2014; Di Virgilio et al., 2017). Moreover, two K+ channels, two-pore domain weak inwardly rectifying K+ channel 2 (TWIK2) and tandem pore domain halothane-inhibited K+ channel 1 (THIK1), have been reported to response to ATP, and regulate NLRP3 inflammasome activation (Di et al., 2018; Drinkall et al., 2022). Subsequently, the triggered K+ efflux can promote NEK7 to interact with NLRP3 *via* the catalytic domain of NEK7 and the LRR domain of NLRP3 (He et al., 2016b). Research has reported that a decrease in cytosolic K+ level triggers the activation of the NLRP3 inflammasome (Muñoz-Planillo et al., 2013). A type of K+ channel inhibitor has also been found to greatly inhibit NLRP3 inflammasome activation (Lamkanfi and Dixit, 2009). Ca2+ fluctuations have a crucial effect on the activation of NLRP3 inflammasome by triggering mitochondrial dysfunction. In detail, influent Ca2+ and the Ca2+ released from the stimulated endoplasmic reticulum can be transported into the mitochondria through uniporters to depolarize the mitochondrial membrane, activating voltage-gated channels of the mitochondria. Subsequent entry of many ions and metabolites trigger the production and release of reactive oxygen species (ROS) (Horng, 2014; Shenker et al., 2015). A recent study demonstrated that the transient receptor potential vanilloid type 1 (TRPV1) channel could regulate the activation of NLRP3 inflammasome by affecting Ca2+ influx. Moreover, its deletion suppressed NLRP3 inflammasome activation, thereby alleviating experimental autoimmune encephalomyelitis (EAE, a common animal model of MS) (Zhang et al., 2021). BAPTA-AM, a Ca2+ chelator, suppresses activation of the NLRP3 inflammasome (Wu et al., 2019). In addition, NLRP3 agonists have been found to induce Ca2+ signaling, triggering NLRP3 inflammasome activation (Murakami et al., 2012). A previous study suggested that the substitution of extracellular Cl- with gluconate increases the maturation and secretion of IL-1β (Verhoef et al., 2005). Levels of cytosolic Cl- were found to be reduced upon NLRP3 inflammasome activation, and inhibition of the Cl- channel could suppress NLRP3 inflammasome activation (Compan et al., 2012; Daniels et al., 2016). Mitochondrial dysfunction plays an important role in the activation of the NLRP3 inflammasome. Research has reported that stimulation of the NLRP3 inflammasome causes mitochondrial dysfunction, which leads to the production and release of mitochondrial ROS (mtROS) and the release of mitochondrial DNA (mtDNA) into the cytoplasm. Subsequently, mtROS can oxidize mtDNA, and mtDNA can be recognized by the NLRP3 inflammasome, leading to its activation (Shimada et al., 2012; Zhong et al., 2018; De Gaetano et al., 2021). Additionally, cardiolipin, which is located in the mitochondria, triggers NLRP3 inflammasome activation (Iyer et al., 2013). Under normal conditions, the thioredoxin interaction protein (TXNIP) and thioredoxin (TRX) bind to each other. Once the level of ROS increases, TRX oxidizes itself to remove ROS, along with the separation of TRX and TXNIP. The separated TXNIP can bind to NLRP3, triggering NLRP3 inflammasome activation. ROS have also been shown to promote the interaction between MINK1 and NLRP3 (Zhu et al., 2021). In addition, the ensuing inflammatory response recruits immune cells to increase ROS production, which suggests a feedback loop between ROS and the NLRP3 inflammasome (Franchi et al., 2009; Dominic et al., 2022). However, a few studies have reported that ROS are not necessary for this activation (Gabelloni et al., 2013; Gurung et al., 2015); therefore, the exact mechanism of mitochondrial dysfunction in the activation of the NLRP3 inflammasome requires further studies. Lysosomal rupture triggers the release of lysosomal contents, thereby triggering NLRP3 inflammasome activation (Halle et al., 2008; Hornung et al., 2008). Among lysosomal contents, cathepsin B is considered important for NLRP3 inflammasome activation. CA-074-Me suppresses NLRP3 inflammasome activation by inhibiting cathepsin B (Hornung et al., 2008). Besides that, it has been reported that ruptured lysosomes could trigger K+ efflux, thus promoting NLRP3 inflammasome activation (Muñoz-Planillo et al., 2013). Moreover, research has demonstrated that stimuli can induce the disassembly of the trans-Golgi network (TGN), and dispersed TGN (dTGN) can recruit NLRP3 proteins *via* ionic bonding between phosphatidylinositol-4-phosphate (PtdIns4P, negatively charged, on the dTGN) and a polybasic region (on the NLRP3 protein), then inducing the assembly and activation of the NLRP3 inflammasome (Chen and Chen, 2018; Shao et al., 2021).

Apart from the canonical activation pathway, NLRP3 inflammasome is also activated *via* non-canonical and alternative pathways (Figure 2). Caspase-11 in mice was found to directly sense cytosolic lipopolysaccharide (LPS) and lead to the formation of pores in the plasma membrane, inducing pyroptosis and K+ efflux, thus triggering non-canonical NLRP3 inflammasome activation without the involvement of caspase-1 (Kayagaki et al., 2011; Hagar et al., 2013; Ramirez et al., 2018). Similarly, studies have demonstrated that caspase-4 and caspase-5 in humans promote non-canonical activation by directly binding to cytosolic LPS (Shi et al., 2014; Casson et al., 2015). Besides that, the involvement of caspase-8 in NLRP3 alternative activation also draws our attention, which is mediated by the TLR4-TRIF-RIPK1-FADD-caspase-8 signaling pathway (Gurung et al., 2014; Gurung and Kanneganti, 2015; Gaidt et al., 2016). Recently, Zhang et al. reported the important role of the caspase-8-dependent inflammasome in CNS inflammation (Zhang et al., 2018).

There is still a lot of unknown information about the mechanisms of NLRP3 inflammasome activation. For example, the temporal and spatial distribution of NLRP3 inflammasome after activation, the contradictory results of major activation signals in NLRP3 inflammasome activation, induction of activation after the generation of major activation signals, and the mechanistic details of non-canonical and alternative activation pathways.

## NLRP3 Inflammasome in the Pathogenesis/Pathophysiology of MS

### Gene Polymorphisms of NLRP3 Signaling Pathway Molecules in MS

The NLRP3 inflammasome has been found to exert important effects in a myriad of diseases (Bonomini et al., 2019; Cheng et al., 2020; Yu et al., 2020). Occurrence of MS is closely related to the NLRP3 inflammasome (Gris et al., 2010; Olcum et al., 2020). Recent research has revealed that alterations in genes of NLRP3-related molecules are associated with susceptibility to MS. A study analyzed the association of SNPs of NLRP3 (rs-10754558, rs-35829419, rs-3806265, rs-4612666) with susceptibility to MS and revealed the pivotal role of NLRP3 polymorphisms in MS (Imani et al., 2018). Moreover, functional genetic variants in NLRP3 (Q705K) are associated with the severity of MS (Soares et al., 2019). In addition to NLRP3 genes, alterations in the gene expression of *PYCARD* and *CASP1* were reported in MS patients who initially presented with the clinically isolated syndrome, which showed an association between them and MS (Hagman et al., 2015). Mutations in the downstream cytokines have also been studied. Research has found that there is no relationship between the polymorphisms of IL-1β−511 (rs16944) and IL-1β +3,953 (rs1143634) and the risk of MS; however, further studies revealed that early-onset MS had a higher association with heterozygosity of rs16944 than with homozygosity of rs16944 in IL-1β (Huang et al., 2013; Isik et al., 2013). Soares et al. analyzed SNPs distribution in different clinical phenotypes of MS and found that variants of−511C>T could result in more frequent in MS (Soares et al., 2019). There are conflicting results regarding the role of IL-18 polymorphisms in MS. Research has revealed that polymorphisms in IL18−607 C/A are not significant, while polymorphisms at position−137 are related to MS risk. However, another study found that polymorphisms of the IL-18−137C/G gene were insignificant, while IL-18−607C/A gene polymorphisms were a potential genetic risk factor for susceptibility to MS (Karakas Celik et al., 2014; Orhan et al., 2016). A recent study analyzed gene polymorphisms of IL-18 and reported that the genotype of rs1946518 was markedly different between MS patients and the control group (Jahanbani-Ardakani et al., 2019).

### NLRP3 Inflammasome-Related Molecules in MS

NLRP3 inflammasome-related molecules are involved in the pathogenesis of MS. Studies have reported increased expression levels of NLRP3 and IL-1β genes in MS plaques and elevated levels of ASC, caspase-1, and IL-18 in the sera of MS patients (Keane et al., 2018; Voet et al., 2018). In EAE, the expression levels of NLRP3 mRNA and protein were increased (Gris et al., 2010; Ke et al., 2017), and compared to the wild-type mice, *Nlrp3*^−/−^ mice showed reduced numbers of Th1 and Th17 cells in the spinal cord and peripheral lymphoid tissues and markedly mild EAE (Gris et al., 2010; Inoue et al., 2012a). Recently, it was reported that thymic stromal lymphopoietin (TSLP) could directly induce NLRP3 expression through phosphorylation of Janus kinase (JAK) 2, and *Tslpr*^−/−^ mice showed decreased NLRP3 expression and EAE scores (Yu et al., 2021). Zhang et al. (2018) reported that a deficiency of microglial ASC could attenuate the expansion of T cells and the infiltration of neutrophil, which suggests the important role of ASC in EAE. In addition, the expression of caspase-1 was found to increase at the mRNA and protein levels (Ke et al., 2017; Bai et al., 2018), and inhibition of caspase-1 could suppress inflammasomes activation, thus attenuating the severity of EAE (Ahmed et al., 2002; McKenzie et al., 2018). Furthermore, a previous study analyzed the severity of EAE in *Asc*^−/−^ and *caspase-1*^−/−^ models and demonstrated that *Asc*^−/−^ mice showed a decreased number of MOG-specific T cells in the lymph nodes, delayed disease progression, and reduced clinical scores (Shaw et al., 2010). In addition, *Asc*^−/−^ mice were more protected from EAE progression than *caspase-1*^−/−^ mice.

### IL-1β and IL-18 in MS

**Figure 3:**
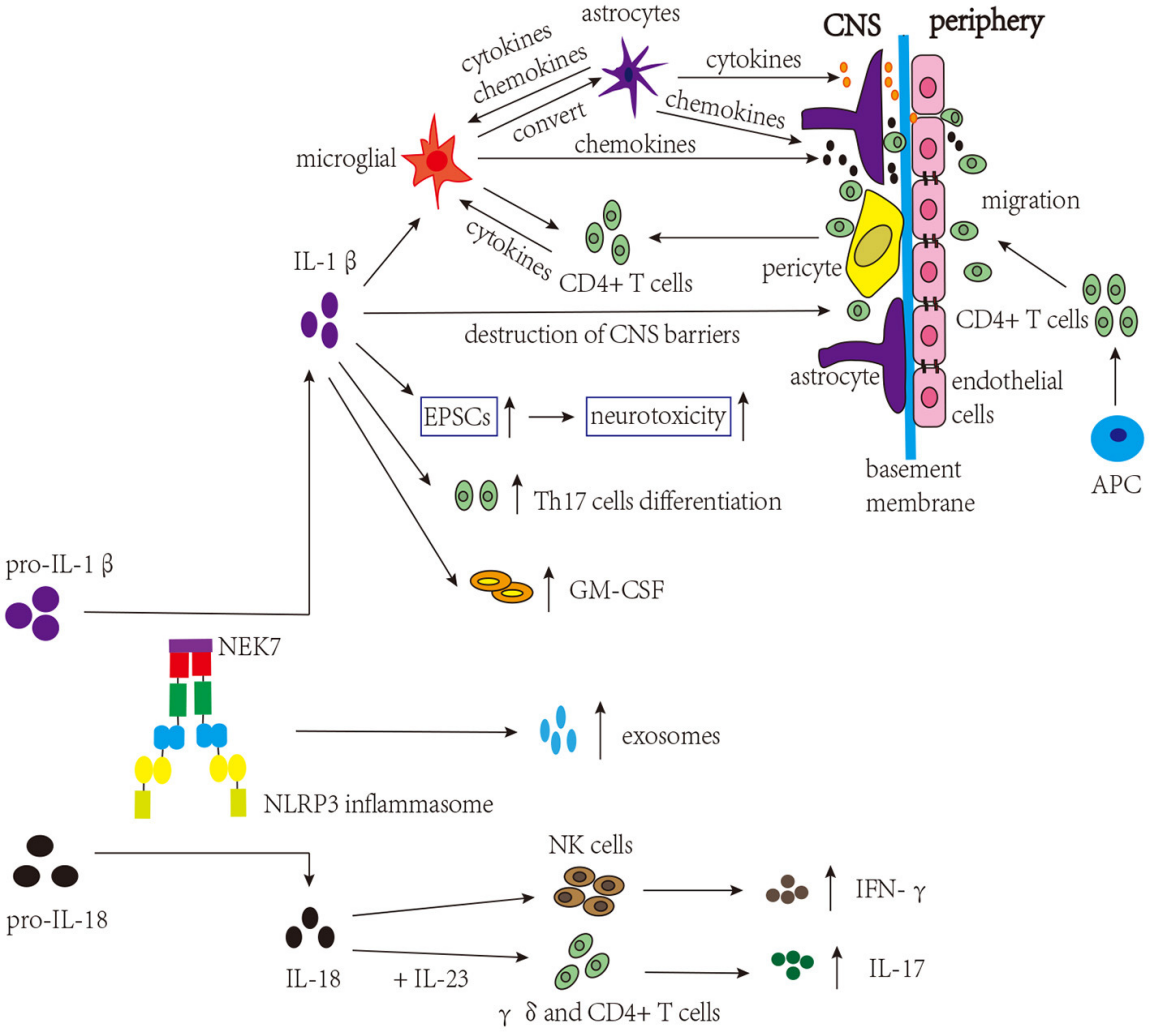
NLRP3 inflammasome-related molecules are involved in the pathogenesis of MS. The NLRP3 inflammasome induces the migration of CD4+ T cells into the CNS and release of exosomes. IL-1β activates microglia, which present self-antigens to activate infiltrated CD4+ T cells, thereby amplifying neuroinflammation. Microglia can convert astrocytes to cytotoxic A1 phenotype and release chemokines to recruit CD4+ T cells. Activated astrocytes release proinflammatory cytokines and chemokines, affecting microglia, tight junctions of endothelial cells, and the infiltration of CD4+ T cells. IL-1β promotes the destruction of CNS barriers, facilitating the infiltration of CD4+ T cells. IL-1β can also increase the levels of EPSCs, contributing to the severity of MS. Moreover, IL-1β can increase the expression of GM-CSF and trigger the differentiation of Th17 cells, which are involved in the pathogenesis of MS. In addition, combined with IL-23, IL-18 can trigger γδ and CD4 + T cells to release innate IL-17, amplifying autoimmunity. IL-18 can also induce the production of IFN-γ by NK cells.

Many studies have focused on the involvement of IL-1β and IL-18 in MS (Figure 3). As mentioned above, in the early stage of MS, the loss of integrity of CNS barriers can cause the infiltration of peripheral immune cells, and IL-1β promotes the destruction of CNS barriers (Kermode et al., 1990; Paul and Bolton, 1995; Paré et al., 2018). Briefly, immune mediators cause dysregulation of junctional components (tight junctions and adherens junctions) of CNS barriers, and the secretion of proinflammatory cytokines further triggers the infiltration of peripheral immune cells (Alvarez et al., 2011). Both CNS astrocytes and endothelial cells are involved in this process, and activated astrocytes can release proinflammatory cytokines that damage the tight junctions of endothelial cells. In addition, chemokines released by activated astrocytes can recruit leukocytes into the CNS, which is involved in the development of MS (Dong and Benveniste, 2001; Ching et al., 2007; Argaw et al., 2012). These proinflammatory cytokines and chemokines can also activate microglia (Correale and Farez, 2015). Microglia, which can be activated by IL-1β, are considered to be a kind of APCs that activate infiltrated CD4+ T cells, amplifying neuroinflammation. Besides that, microglia secrete chemokines that recruit immune cells. In turn, infiltrated CD4+ T cells secrete proinflammatory cytokines to activate microglia (Ferrari et al., 2004; Mallucci et al., 2015). According to existing research, microglia, astrocyte, and CD4+ T cells are the major cell types involved in NLRP3 inflammasome activation in MS. Studies have shown that the proportion of microglia expressing NLRP3 and IL-1β is positively associated with the degree of demyelination in MS patients (Malhotra et al., 2020). Furthermore, after activation, the NLRP3 inflammasome in activated microglia converts astrocytes to the neurotoxic A1 phenotype in MS, aggravating cognitive deficits (Hou B. et al., 2020). Tibolone and sinomenine were reported to alleviate astrocytic and microglial reactions and mobilization, respectively, decreasing NLRP3 inflammasome activation and EAE severity, which further supports the pathogenic role of the NLRP3 inflammasome in MS (Kiasalari et al., 2021; Mancino et al., 2022). Excitatory neurotransmitters can induce excitatory postsynaptic currents (EPSCs), which promote neurotoxicity and are involved in MS. IL-1β can increase the level of EPSCs in corticostriatal slice cultures and cerebellum and contribute to the severity of EAE (Gentile et al., 2013; Mandolesi et al., 2013). In addition, it was found that IL-1β could promote the expression of granulocyte-macrophage colony-stimulating factor (GM-CSF) and the differentiation of Th17 cells, thus being involved in the pathogenesis of MS (Chung et al., 2009; Russi et al., 2018). However, a previous study found elevated levels of the IL-1β protein only in the spinal cord but not in the brain (McKenzie et al., 2018). In addition to IL-1β, the expression level of the IL-18 mRNA was increased in the CNS during EAE, and increased IL-18 levels could aggravate demyelination and neurodegeneration in EAE (Jander and Stoll, 1998; Shi et al., 2000; Jha et al., 2010). IL-18 triggers γδ and CD4+ T cells to release innate IL-17, thereby amplyfing autoimmunity (Lalor et al., 2011). Moreover, research has demonstrated that *Il-18*^−/−^ mice are resistant to EAE, and the functions of natural killer (NK) cells and the production of autoreactive Th1 cells are impaired (Shi et al., 2000). Although studies on MS and EAE have shown the important role for IL-18 in the disease, the results are still conflicting. In one study, the symptoms of *Il-18*^−/−^ mice were significantly alleviated, while in another study, the alleviation was not obvious, which may be related to the method of disease induction and generation of animal models (Gutcher et al., 2006; Gris et al., 2010). These studies indicate that IL-1β and IL-18 participate in the course of MS; however, further studies are needed to understand the exact mechanisms and target them for the treatment of MS.

### NLRP3 Inflammasome and T Cells in MS

Research has demonstrated that the change in the population of T cells is perhaps not important in MS, whereas their migration into the CNS, induced by NLRP3 inflammasome, mediates the development of MS (Inoue et al., 2012a,b). As an important component of innate immunity, the NLRP3 inflammasome connects innate and adaptive immunity by promoting the production of IL-1β and IL-18, increasing the infiltration of peripheral immune cells into the CNS, and affecting the function of T and B cells. A previous study has proposed that Th cells need to be primed by APCs containing NLRP3 inflammasome before their migration into the CNS, and the study found several related results: (1). The number of immune cells migrating into the CNS was reduced in *Asc*^−/−^ and *Nlrp3*^−/−^ mice. (2). The expression levels of migration-related genes were increased by the NLRP3 inflammasome in Th cells and APCs. More specifically, increased levels of OPN, CCR2, and CXCR6 were found in Th cells, and the expression of α4β1 integrin, CCL2, CCL7, CCL8, and CXCL16 was increased in APCs. Interestingly, these components can bind with matched ligands or receptors (OPN-α4β1 integrin; CCR2-CCL2, CCL7, CCL8, and CXCL16; CXCR6-CXCL16) (Inoue et al., 2012a). Therefore, targeting immune cell migration is probably an effective treatment for MS. After migration, primed T cells play an important role in the course of the disease, and the NLRP3 inflammasome, in both T cells and microglia, has been reported to induce T cells to release proinflammatory cytokines involved in the pathogenesis of MS (Inoue et al., 2012a; Olcum et al., 2020).

### NLRP3 Inflammasome and B Cells in MS

Although most studies suggest that MS is a kind of immune disease predominantly involving T cells, growing research has demonstrated that B cells are also crucial in the development of the disease (Li et al., 2018). The mechanism of action of B cells in MS includes four aspects: antigen presentation, cytokine production, antibody production, and ectopic lymphogenesis (Lazibat et al., 2018). B cells can present antigens to T cells, which is important for T cells activation, and the interaction between B and T cells is direct and two-way (Dalakas, 2008; Lazibat et al., 2018). Moreover, cytokines produced by B cells also exert effects on MS; for instance, B cells increase the expression of IL-6, activating Th17 responses in MS (Li et al., 2018). There are no antibodies in the CNS of healthy individuals; therefore, the presence of antibodies in the cerebrospinal fluid (CSF) of MS patients suggests that B cells are involved in the development of MS. Immunoglobulin G (IgG) and immunoglobulin M (IgM) oligoclonal bands could be used as biomarkers of MS and to predict the course of MS (Villar et al., 2005). Ectopic lymphogenesis refers to the formation of ectopic lymphoid structures, which are similar to follicles in MS patients and contain B cells; they are associated with EBV infection and may be involved in the development of MS (Serafini et al., 2004). Research has demonstrated a relationship between NLRP3 inflammasome and B cells. A recent study has reported that B cell-activating factor (BAFF) activates the NLRP3 inflammasome in B cells by triggering the binding of cIAP-TRAF2 to components of the NLRP3 inflammasome and by promoting K+ efflux and production of Src activity-dependent ROS (Lim et al., 2020). β-glucan and CpG, two major microbial antigens, have been reported to trigger NLRP3 inflammasome activation in circulating B cells. It was found that Dectin-1 activation *via* SYK and mammalian target of rapamycin (mTOR) participates in this process (Ali et al., 2017). Although there are many studies on the relationship between NLRP3 inflammasome and B cells, their relationship in MS is not clear, and further studies are needed to clarify the underlying mechanism.

### NLRP3 Inflammasome and Exosomes in MS

In recent years, increasing attention has been paid to the role of exosomes, in tumors and autoimmune diseases, as important messengers in cell-to-cell communication (Prada et al., 2013; Paolicelli et al., 2019). It was revealed that exosomes could be selectively loaded with immunomodulatory proteins, which make them vital media for transmitting biological signals (Cypryk et al., 2018). Studies have found that damage to the CNS can induce inflammasomes activation, and exosome secretion is related to inflammasome activity. Therefore, exosomes induced by inflammasomes can promote the inflammatory response in recipient cells by transporting inflammasome-related components and other substances (de Rivero Vaccari et al., 2016; Hezel et al., 2017; Zhang et al., 2017). Zhang et al. (2017) found that inflammasome-derived exosomes could activate the NF-kB signaling pathway in recipient cells, thereby promoting an inflammatory response. Furthermore, recent research has suggested that NLRP3 inflammasome activation in microglia could promote the secretion of exosomes and facilitate the transmission of α-synuclein in Parkinson's Disease (PD) (Si et al., 2021). However, a recent study found that exosomes loaded protective molecules can protect against cardiovascular damage (Pearce et al., 2021). The above studies indicate that exosomes may be protective or damaging, which is closely related to the environment and condition of the cells from which they originate. Besides that, the effects of exosomes on inflammasomes are also being studied. Research has demonstrated that exosomes/microvesicles from periodontal ligament stem cells of MS patients can inhibit NLRP3 inflammasome activation and attenuate the severity of EAE (Soundara Rajan et al., 2017). In detail, the effects of exosomes on NLRP3 inflammasome activation depend on the origin of exosomes, including inhibition (from plasma, mesenchymal stem cells, and macrophages) and promotion (from cancer cells) (Li et al., 2021). For example, Yan et al. (2020) reported that exosomes derived from human umbilical cord mesenchymal stem cells could inhibit NLRP3 inflammasome activation by releasing circular RNA homeodomain-interacting protein kinase 3 (circHIPK3). In addition, cancer-derived exosomes have been found to promote NLRP3 inflammasome activation in macropahges (Liang et al., 2020). However, the relationship between the NLRP3 inflammasome and exosomes in MS is not well-understood, and more studies are needed. Alteration of the components of these exosomes or inhibition of their transfer may be promising therapies for the treatment of MS.

### NLRP3 Inflammasome and Intestinal Flora in MS

The intestinal flora has been a research hotspot in recent years. It develops from birth, is affected by a variety of internal and external factors, and reaches a relatively stable state of composition (Mueller et al., 2015). Recent studies have found that dyregulation of the intestinal flora affects the development of many diseases, including CNS disorders (Ochoa-Repáraz et al., 2009; Sommer and Bäckhed, 2013). This suggests the presence of communication between the intestine and CNS, which we refer to as gut-brain axis (GBA). GBA is a complicated bidirectional communication among the microbiota, gut and CNS, involving the microbiota and its metabolites, immune system, endocrine system, and nervous system (Rutsch et al., 2020). Gut microbiota can act on intestinal epithelial cells to regulate their growth and differentiation, expression of tight junction proteins, and permeability of mucous membranes, thereby maintaining the integrity of the intestinal epithelial barrier. Furthermore, the release of effector molecules following activation of the intestinal inflammasome by the intestinal flora can affect the CNS *via* the vagus nerve (Sharma et al., 2010; Rao et al., 2017). Many studies have suggested a relationship between intestinal flora and MS. The composition of intestinal flora in MS patients is different from that in healthy individuals. Studies have demonstrated that the abundance of Bacteroides decreased and that of Pseudomonas increased in the intestinal flora of MS patients, while the protein levels of NLRP3, ASC and cleaved caspase-1 increased in the CNS, and the mRNA levels of ASC and caspase-1 increased in the circulation (Pellegrini et al., 2020). It has been reported that overactivation of the NLRP3 inflammasome can promote the shift of intestinal flora to a proinflammatory phenotype, which in turn promotes neuroinflammation and neurodegeneration (Pellegrini et al., 2020). Mackay et al. reported that consumption of a high-fiber diet, which affects the host microbiota, increases the levels of IL-18 in the serum through GPR43 and GPR109A receptors, which are expressed on colonic epithelial cells, and trigger K+ efflux and Ca2+ mobilization in an NLRP3-dependent manner (Macia et al., 2015). Mechanistically, certain metabolites produced by the microbiota in the body can enter the bloodstream and directly affect peripheral immune cells. Alternatively, these metabolites can enter the brain through the bloodstream and affect immune cells in the CNS (Wikoff et al., 2009; Rothhammer et al., 2016), which is regulated by the NLRP3 inflammasome as an important component that bridges innate and adaptive immunity. However, the exact mechanism of interplay between the microbiota and NLRP3 inflammasome is currently unknown, as is the mechanism of the subsequent effects of this interaction on the CNS. A study proposed that the effect of the interaction between the microbiota and NLRP3 inflammasome on the CNS may be through regulation of the plasma level of the circHIPK2, which in turn affects astrocyte function (Zhang et al., 2019). Furthermore, the existence of GBA is further supported by a study showing improvement in severe constipation and other MS symptoms after fecal microbiota transplantation in three MS patients (Rutsch et al., 2020). Besides that, the use of probiotics or antibiotic cocktails could lead to attenuation of EAE (Colpitts et al., 2017; Camara-Lemarroy et al., 2018). Pertussis toxin is required for the induction of EAE and is a major virulence factor of *Bordetella pertussis*. A study demonstrated that pertussis toxin could trigger the activation of TLR4 and induce the formation of a pyrin-dependent inflammasome, which only leads to mild EAE in pyrin-, ASC-, or caspase-1-deficient models (Dumas et al., 2014). The above studies have shown a connection among intestinal flora, inflammasomes, and MS; however, the underlying mechanism is still unclear, and further research is needed to clarify the molecules, pathways, and organs involved. This will be an attractive research area for the development of MS treatment in the future.

## NLRP3 Inflammasome as a Biomarker in MS

Studies have shown that NLRP3 inflammasome-related components can be used as biomarkers for MS. For example, NLRP3 protein was found to be overexpressed in the monocytes of MS (Malhotra et al., 2020) and neuromyelitis optica spectrum disorders patients (Peng et al., 2019), compared to those of healthy individuals. In addition, elevated levels of ASC and caspase-1 were found in the sera of MS patients, and ASC was considered to be more sensitive and specific for the diagnosis of MS severity owing to its 90% specificity (Keane et al., 2018). A recent study showed that serum caspase-1 levels were elevated during relapse in MS patients, suggesting that caspase-1 may act as a biomarker of MS disease activity (Beheshti et al., 2020).

As the downstream effectors of the NLRP3 inflammasome, IL-1β and IL-18 can be used as potential biomarkers for MS. The results of studies on the expression level of IL-1β in the sera and CSF of MS patients are contradictory, and the level of IL-1β was increased or not detected in different studies (Maimone et al., 1991; Peter et al., 1991; Dujmovic et al., 2009). However, the expression of IL-1β in the CSF is associated with cortical pathology in the early stage of MS and causes neurodegeneration, following neuroinflammation (Seppi et al., 2014). One study reported increased serum IL-18 levels in MS patients, and several other studies also reported increase in IL-18 levels in the sera and CSF of MS patients (Losy and Niezgoda, 2001; Chen et al., 2012; Jahanbani-Ardakani et al., 2019).

In recent years, the neurofilament protein has been considered a promising biomarker for MS. The neurofilament protein is a cytoskeletal protein expressed in neurons, and it can be released into the blood and CSF after the damage of neurons (Khalil et al., 2018; van Lieverloo et al., 2019). Serum neurofilament protein is a useful biomarker for progressive MS and pediatric MS, and its level is associated with the risk of disability in MS (Kapoor et al., 2020; Manouchehrinia et al., 2020; Reinert et al., 2020). It consists of three isotypes: a neurofilament light (NfL) chain, neurofilament intermediate (NfM) chain, and neurofilament heavy (NfH) chain. Elevated NfL levels in the CSF occur in all stages of MS, and their levels are correlated with the MS severity score (Salzer et al., 2010; Teunissen and Khalil, 2012). In addition, interleukin-1 receptor antagonist (IL-1RA) was considered a novel biomarker in MS patients and was associated with NfL in a separate cohort (Blandford et al., 2021). Neurofilament degradation is one of the main pathological damage caused by chronic exposure to *n*-hexane in humans (Huang, 2008; Wang et al., 2008). A recent study found that glibenclamide ameliorated 2,5-hexanedione (the toxic metabolite of *n*-hexane)-induced axon degeneration by inhibiting NLRP3 inflammasome activation, thus suppressing NfL reduction in rats (Hou L. et al., 2020). This suggests a relationship between the NLRP3 inflammasome and NfL; however, their exact relationship in MS is not fully understood, and further studies are needed.

## Targeting NLRP3 Inflammasome-Related Pathways for the Treatment of MS

In this section, we briefly summarize therapies targeting NLRP3 inflammasome-related pathways for the treatment of MS (Table 1).

**Table 1:** Compounds targeting NLRP3 inflammasome-related pathways.

**Classification**	**Target**	**Compound**	**Mechanism**	**References**
Anti-IL-1 therapies	IL-1β	Canakinumab	IL-1β neutralizing antibody	Chakraborty et al., 2012; Dinarello et al., 2012
	IL-1β	Anakinra	IL-1 receptor antagonist	Dinarello et al., 2012; Cavalli and Dinarello, 2018
	IL-1β	Rilonacept	Soluble decoy receptor for IL-1	Dinarello et al., 2012; Abbate et al., 2020
	IL-1β	Cladribine	Decreases IL-1β-induced EPSCs	Musella et al., 2013
Upstream major activation signals	K+	β-hydroxybutyrate	Attenuates K+ efflux and oligomerization of NLRP3 and ASC	Youm et al., 2015
	K+	Probenecid	Blocks Pannexin1, which mediates K+ efflux	Jian et al., 2016
	K+	Brilliant blue G	A P2X7R antagonist, P2X7R is deemed to mediate K+ efflux	Chen et al., 2013
	Ca2+	U73122	A PLC inhibitor, can block oxaliplatin-induced intracellular Ca2+ influx	Potenzieri et al., 2020
	Cl-	IAA-94	blocks Cl- channels in cultured rabbit cardiomyocytes	Diaz et al., 2013
	Mitochondria	N-acetyl-L-cysteine, APDC	ROS inhibitors, decrease the production of IL-1β in THP1 cells	Dostert et al., 2008
	Mitochondria	MitoTEMPOL	An effective antioxidant, prevents lipid peroxidation and protects the mitochondria	Trnka et al., 2009
	Mitochondria	Bixin	Scavenges ROS through the NRF2 signaling pathway, inhibits EAE severity	Yu et al., 2020
	Lysosome	CA-074-Me	Causes selective inactivation of intracellular cathepsin B	Buttle et al., 1992; Hornung et al., 2008
	Lysosome	Gemcitabine, 5-fluorouracil	Induce the release of cathepsin B from lysosomes, thus triggering direct activation of the NLRP3 inflammasome	Bruchard et al., 2013
	Lysosome	Curcumin	Inhibits the release of ROS and cathepsin B, inhibits the progression of EAE	Hasanzadeh et al., 2020; Lu et al., 2020
Upstream regulatory pathways	Autophagy	HU-308	A cannabinoid receptor 2 agonist, induces autophagy	Shao et al., 2014
	Autophagy	Caffeine	Induces autophagy, inhibits NLRP3 inflammasome activation	Wang et al., 2022
	NO	CD47-Fc fusion protein	Increases the production of NO, thus reducing the level of IL-1β	Gao et al., 2016
	Post-translation modification	IFN-β	Induces the phosphorylation of STAT1, attenuating the severity of NLRP3 inflammasome-dependent EAE	Inoue et al., 2012b, 2016; Metwally et al., 2020
NLRP3 inflammasome-related components	NLRP3	MCC950	Binds to Walker B motif of the NACHT domain, inhibits ATPase activity of NLRP3, reduces the severity of EAE	Coll et al., 2019
	NLRP3	Bay11-7082	Binds to ATPase of NLRP3 NACHT domain, inhibits EAE severity	Lang et al., 2021
	NLRP3	Oridonin, RRx-001	Bind to cysteine 279 or 409 of NLRP3 in the NACHT domain, inhibit NEK7-NLRP3 interaction, attenuate EAE severity	He et al., 2018; Yang et al., 2020; Chen et al., 2021
	NLRP3	OLT1177	Inhibits NLRP3-ASC interaction by binding to ATPase of NLRP3 NACHT domain, ameliorates EAE severity	Sánchez-Fernández et al., 2019
	ASC	1,2,4-TTB	Inhibits oligomerization of ASC and NLRP3-ASC interaction, thus decreasing EAE scores	Pan et al., 2021
	ASC	IC100	Targets ASC and inhibits EAE progression	Desu et al., 2020
	Caspase-1	Ac-YVAD-CMK	A caspase-1 inhibitor, reduces the mRNA and protein levels of IL-1β	Mao et al., 2017
	Caspase-1	VX-765	A caspase-1 inhibitor, covalently modifies Cys285 of caspase-1	McKenzie et al., 2018
	GSDMD	Disulfiram	Covalently modifies Cys191/Cys192 in GSDMD, upregulates miR-30a expression in EAE	Zhao et al., 2016; Hu et al., 2020

### Anti-IL-1 Therapies

IL-1β contributes to the progression of MS. Related therapies that have been studied to date include IL-1β-specific neutralizing antibody (canakinumab), IL-1 receptor antagonist (anakinra), and soluble decoy receptor for IL-1α and IL-1β (rilonacept), and the first two have been used in clinical practice (Chakraborty et al., 2012; Dinarello et al., 2012; Cavalli and Dinarello, 2018; Abbate et al., 2020). In addition, studies have demonstrated that cladribine can attenuate IL-1β-induced EPSCs and is beneficial for the treatment of MS (Musella et al., 2013). However, the use of anti-IL-1 therapies is costly, and their penetration across brain tissues is poor. Moreover, IL-1β is not the only downstream cytokine of the NLRP3 inflammasome; therefore, targeting upstream molecules of the NLRP3 inflammasome is perhaps more effective than anti-IL-1 therapies.

### Targeting Upstream Major Activation Signals

It has been reported that β-hydroxybutyrate can attenuate K+ efflux and suppress oligomerization of NLRP3 and ASC, thus inhibiting NLRP3 inflammasome activation (Youm et al., 2015). Pannexin1 and P2X7R mediate K+ efflux. Probenecid, which blocks Pannexin1, attenuates NLRP3 inflammasome activation (Jian et al., 2016). Brilliant blue G is a P2X7R antagonist, and it suppresses neuroinflammation in subarachnoid hemorrhage animal models, which may be associated with the inhibition of K+ efflux (Chen et al., 2013). Therapies targeting Ca2+ and Cl- have also been studied, including U73122 and IAA-94 (Diaz et al., 2013; Potenzieri et al., 2020). However, therapies targeting ion fluctuations can produce unavoidable side effects caused by the dysregulation of ion-related reactions. Therefore, although therapies targeting ion fluctuations are effective in the experiment, their usage in clinical practice may be limited. ROS scavengers, including N-acetyl-L-cysteine (2R,4R)-4-aminopyrro lidine-2,4-dicarboxylate (APDC), and Mito-TEMPO, were found to inhibit ROS production and attenuate NLRP3 inflammasome activation (Dostert et al., 2008; Trnka et al., 2009). In addition, Yu et al. found that Bixin could scavenge ROS through the NRF2 signaling pathway and attenuate the severity of EAE (Yu et al., 2020). Studies have suggested that mtROS can damage NADPH oxidase, causing inflammasome activation (Dostert et al., 2008; Bordt and Polster, 2014). However, similar to ion fluctuations, mtROS are also involved in many biological reactions; thus, the inhibition of mtROS cause side effects, and therapies targeting mtROS require further study. It has been reported that cathepsin B released from lysosomes plays a role in NLRP3 inflammasome activation, and CA-074-Me attenuates NLRP3 inflammasome activation by inhibiting cathepsin B (Buttle et al., 1992; Hornung et al., 2008). In addition, gemcitabine and 5-fluorouracil have been shown to contribute to the activation of NLRP3 inflammasome, which is related to cathepsin B (Bruchard et al., 2013). Recently, research has demonstrated that curcumin can inhibit the release of ROS and cathepsin B, thereby inhibiting the progression of EAE (Hasanzadeh et al., 2020; Lu et al., 2020).

### Targeting Upstream Regulatory Pathways

In addition to major activation signals, other regulatory mechanisms are involved in NLRP3 inflammasome activation. HU-308, a cannabinoid receptor 2 agonist, was reported to induce autophagy, thus attenuating NLRP3 inflammasome activation and severity of EAE (Shao et al., 2014). Moreover, a recent study found that caffeine can reduce NLRP3 inflammasome activation by inducing autophagy, thus attenuating the severity of EAE (Wang et al., 2022). It was found that the CD47-Fc fusion protein could reduce the production of NLRP3 inflammasome-induced IL-1β by promoting the production of NO, aiding to the treatment of EAE (Gao et al., 2016). In addition, as a first-line drug in the treatment of MS, IFN-β can induce the phosphorylation of STAT1, which mainly exerts therapeutic effects on NLRP3 inflammasome-dependent EAE (Inoue et al., 2012b, 2016; Metwally et al., 2020). However, the reactions involved in these regulatory mechanisms are more complicated, and side effects will inevitably occur, which means that therapies targeting molecules upstream of the NLRP3 inflammasome may not be as potent as directly targeting NLRP3 inflammasome-related components.

### Targeting NLRP3 Inflammasome-Related Components

MCC950, a classic NLRP3 inhibitor, can bind to the Walker B motif of the NACHT domain of NLRP3 and inhibit ATPase activity, thereby reducing the severity of EAE (Coll et al., 2019). Furthermore, Bay11-7082 was found to interact with the ATPase of the NLRP3 NACHT domain and reduce EAE severity (Lang et al., 2021). The NEK7-NLRP3 interaction is important for activation of the NLRP3 inflammasome. Research has demonstrated that oridonin and RRx-001 can inhibit this interaction by binding to cysteine 279 of the NACHT domain of NLRP3 or covalently binding to cysteine 409 of NLRP3, thereby attenuating EAE severity (He et al., 2018; Yang et al., 2020; Chen et al., 2021). Besides that, it was reported that OLT1177 could interact with the ATPase of the NLRP3 NACHT domain, inhibiting NLRP3-ASC interaction and ameliorating EAE severity (Sánchez-Fernández et al., 2019). 1,2,4-TTB was found to inhibit the oligomerization of ASC and NLRP3-ASC interactions and reduce EAE scores (Pan et al., 2021). Recently, IC100 has been reported to target ASC and inhibit EAE progression (Desu et al., 2020). Caspase-1 is an effector of NLRP3 inflammasome activation, and several caspase-1 inhibitors have been reported. For example, Ac-YVAD-CMK, a caspase-1 inhibitors, decreases the mRNA and protein levels of IL-1β and improves the symptoms of PD models (Mao et al., 2017). Moreover, VX-765 can covalently modify Cys285 of caspase-1 for the treatment of inflammatory diseases (McKenzie et al., 2018). Research has reported that the level of GSDMD, which is crucial in pyroptosis, can be inhibited by disulfiram by upregulating miR-30a expression (Zhao et al., 2016). A recent study further showed that disulfiram covalently modified Cys191/Cys192 in GSDMD, thus blocking pore formation (Hu et al., 2020).

Taken together, there are many studies on therapies targeting NLRP3 inflammasome-related pathways. However, only a few drugs can be used clinically, and there are no drugs that can effectively cure MS. Therefore, further research is necessary, and studies on the NLRP3 inflammasome-related pathway can shed light on the treatment of MS patients.

## The Involvement of the NLRP3 Inflammasome in Neurodegenerative Diseases

In addition to MS, the NLRP3 inflammasome has important roles in neurodegenerative diseases such as Alzheimer's disease (AD), PD, and amyotrophic lateral sclerosis (ALS).

### NLRP3 Inflammasome in AD

AD is a neurodegenerative disease with a very high prevalence, characterized by progressive loss of neurons and accumulation of extracellular amyloid beta (Aβ) (Piancone et al., 2021). The clinical manifestations of AD include dementia and cognitive dysfunction, which imposes a large burden on patients and their families (Swanton et al., 2018). Currently, the pathogenesis of AD is thought to be associated with the presence of amyloid plaques and neurofibrillary tangles, while neuroinflammation has also been found to be involved (DeTure and Dickson, 2019). Growing evidence suggests that the NLRP3 inflammasome is involved in the pathogenesis of AD. In 2008, a study reported that fibrillar Aβ could promote NLRP3 inflammasome activation, which was confirmed in subsequent studies (Halle et al., 2008). For example, amylin receptors participate in Aβ-induced NLRP3 inflammasome activation. Furthermore, Aβ can increase the level of ROS, which trigger NLRP3 inflammasome activation (Fu et al., 2017; Aminzadeh et al., 2018). Clinically, elevated expression levels of active caspase-1 and IL-1β can be found in AD patients (Blum-Degen et al., 1995; Heneka et al., 2013), which is related to the activation of the NLRP3 inflammasome. Further mechanistic studies were performed in the AD models. A study found that the levels of neuroinflammation and Aβ accumulation were reduced with concomitant improvement of neuronal function in NLRP3-deficient AD models (Heneka, 2017). Besides that, deletion of NLRP3 or caspase-1 in AD models was shown to decrease the levels of IL-1β and caspase-1 while promoting microglial differentiation to the M2 phenotype (Dempsey et al., 2017). The above studies have demonstrated the role of NLRP3 inflammasome activation in AD, but the underlying mechanisms still need to be explored.

### NLRP3 Inflammasome in PD

PD is a common neurodegenerative disease characterized by a progressive loss of dopaminergic (DA) neurons in the substantia nigra and the formation of intracellular Lewy bodies by α-synuclein (Petrucci et al., 2014). The clinical manifestations of PD include typical motor symptoms and other non-motor symptoms; however, the underlying etiology is currently not clear. A previous study suggested the important role of the NLRP3 inflammasome in the pathogenesis of PD *in vitro* (Codolo et al., 2013), which is supported by a growing number of studies. In animal models, the levels of NLRP3 inflammasome-related molecules are increased, while deletion of NLRP3 could reduce the loss of DA neurons and improve motor dysfunction (Qiao et al., 2018; Lee et al., 2019). The involvement of the NLRP3 inflammasome in the pathogenesis of PD has also been reported in PD patients. A study reported increased expression levels of NLRP3 inflammasome-related molecules in PD patients; however, in another study, it was found that although there was a difference in the expression level of NLRP3 in the sera of PD patients compared to those of controls, the levels of IL-1β and IL-18 were not significantly different (Zhang et al., 2016; Fan et al., 2020). Considering the important role of α-synuclein in the pathogenesis of PD, several studies have explored the relationship between α-synuclein and the NLRP3 inflammasome. Research has shown that α-synuclein can promote NLRP3 inflammasome activation in PD models, and further studies have revealed that α-synuclein increases the levels of cathepsin B and ROS, thus triggering NLRP3 inflammasome activation (Codolo et al., 2013; Chatterjee et al., 2020). Further studies are needed to explore the underlying mechanisms of the NLRP3 inflammasome in the pathogenesis of PD, which may shed light on the treatment of PD patients.

### NLRP3 Inflammasome in ALS

ALS, a prevalent neurodegenerative disease, is characterized by the loss of motor neurons (Feng et al., 2021). ALS patients clinically present with a continuous aggravation of motor function loss until death. Mutations in Cu/Zn superoxide dismutase 1 (SOD1) have been found to be associated with ALS (Rosen et al., 1993). The etiology of ALS remains unclear, but the role of neuroinflammation and the NLRP3 inflammasome in ALS has received attention in recent years. Studies have found increased levels of NLRP3 and caspase-1 in astrocytes and the brains of ALS patients (Haidet-Phillips et al., 2011; Kadhim et al., 2016). Studies have used G93A-*SOD1* mutation models to simulate the pathogenesis of ALS. Research has revealed increased levels of NLRP3 inflammasome-related molecules and IL-1β in models, and genes knockout of caspase-1 and IL-1β can inhibit neuroinflammation (Johann et al., 2015; Lall and Baloh, 2017). Further studies are needed to explain these results and clarify the pathogenesis of ALS.

## Conclusions

MS is an immune-mediated, demyelinating, neurodegenerative disease that occurs in the CNS. It predominantly affects young women and causes a huge social and economic burden. Nowadays, the underlying mechanism of MS is not fully understood, and there are still some limitations in the related diagnostics methods and therapies. In recent years, the role of inflammasomes in MS has received widespread attention. For the most widely studied NLRP3 inflammasome, we briefly introduced its composition, mechanisms of activation, and related research in MS, which may help us better understand its involvement in MS.

However, there are several unclear aspects of the NLRP3 inflammasome. For example, NLRP3 inflammasome activation is widely regulated, and it is necessary to clarify each step of the cascade of activation. Although there is a close relationship between the NLRP3 inflammasome and EAE, NLRP3 inflammasome-independent EAE also exists (Arnason, 1999). For example, 300 μg heat-killed mycobacteria was found to successfully induce EAE in *Asc*^−/−^ or *Nlrp3*^−/−^ animal models, while normal 200 μg Mtb mycobacteria could not (Inoue et al., 2012b). In addition, a previous study reported that aggressive immunization induces EAE in *caspase-1*^−/−^ mice (Furlan et al., 1999). Therefore, the intensity of immunization has a great influence on the induction of EAE, which also suggests the important role of environmental factors in the heterogeneity of MS. A study proposed a possible mechanism for the development of NLRP3 inflammasome-independent EAE, which involves membrane-bound lymphotoxin-β receptor (LTβR) and CXC chemokine receptor 2 (CXCR2), but the exact mechanism is yet to be studied in depth (Inoue et al., 2016). Besides that, studies have shown that IFN-β, a long-used clinical first-line drug, is not therapeutically effective for some types of MS and EAE. Further mechanistic studies have identified IFN-β to have a therapeutic effect on NLRP3-dependent EAE models, which also reflects the heterogeneity of MS (Inoue et al., 2012b). Moreover, EAE mainly simulates MS through immune induction; however, there is no such artificial induction method of MS in patients which can fully simulate the heterogeneity and complexity of MS. Therefore, new and better animal models need to be developed. At present, relevant research on the role of the NLRP3 inflammasome in MS is mainly at the level of cells and animal models, and has not yet involved humans. Therefore, further in-depth studies are urgently required.

## Author Contributions

JF was responsible to conceive, design, and revise this review. YC wrote the original manuscript. HY and ZB completed the figures and tables. LW and LY searched and collected the related references. All authors contributed to the article and approved the submitted version.

## Funding

This work was supported by the Outstanding Scientific Fund of Shengjing Hospital (to JF), Key Research and Development Program of Liaoning Province (No. 2020JH2/10300047 to JF), and National Natural Science Foundation of China (No. 81771271 to JF).

## Conflict of Interest

The authors declare that the research was conducted in the absence of any commercial or financial relationships that could be construed as a potential conflict of interest. The reviewer TH declared a shared parent affiliation with the authors to the handling editor at the time of review.

## Publisher's Note

All claims expressed in this article are solely those of the authors and do not necessarily represent those of their affiliated organizations, or those of the publisher, the editors and the reviewers. Any product that may be evaluated in this article, or claim that may be made by its manufacturer, is not guaranteed or endorsed by the publisher.
